# Upregulation of flavin-containing monooxygenase 3 mimics calorie restriction to retard liver aging by inducing autophagy

**DOI:** 10.18632/aging.102666

**Published:** 2020-01-11

**Authors:** Donghao Guo, Yun Shen, Wei Li, Qinjie Li, Ya Miao, Yuan Zhong

**Affiliations:** 1 Department of Geriatrics, Shanghai Jiao Tong University Affiliated Sixth People’s Hospital, Shanghai, China; 2 Department of Medicine and Therapeutics, Faculty of Medicine, The Chinese University of Hong Kong, Shatin, Hong Kong

**Keywords:** flavin-containing monooxygenase 3 (FMO3), calorie restriction (CR), anti-aging, autophagy, mammalian target of rapamycin (mTOR)

## Abstract

Flavin-containing monooxygenase 3 (FMO3) gene expression is often upregulated in long-lived murine models. However, the specific relationship between FMO3 and aging remains unknown. Here, we show that 40% calorie restriction (CR), which is considered to be one of the most robust interventions to delay aging progression, markedly upregulates FMO3. Most importantly, upregulation of hepatocyte FMO3 in murine models prevented or reversed hepatic aging. Accordingly, the upregulation of FMO3 mimicked the effects of CR: reduced serum levels of pro-inflammatory cytokine interleukin-6 and fasting insulin; relief of oxidative stress, with lower hepatic malondialdehyde levels and higher superoxide dismutase activity; reduced serum and hepatic levels of total cholesterol and triglyceride, as well as reduced lipid deposition in the liver; and diminished levels of aging-related markers β-gal and p16. There were also synergistic effects between FMO3 upregulation and CR. Inhibition of autophagy blocked the anti-aging effects of upregulation of hepatocyte FMO3, including reversing the amelioration of the serum and hepatic parameters related to inflammation, oxidative stress, lipid metabolism, liver function, and hepatocyte senescence. Our results suggest that the upregulation of FMO3 mimics CR to prevent or reverse hepatic aging by promoting autophagy.

## INTRODUCTION

Aging is a time-dependent deteriorative process of cells, tissues, and organs, leading to impairment of their structure and functional capacities [1, 2]. Although the liver has great regeneration capacity [3], studies have demonstrated that aging is associated with gradual alteration of hepatic structure and function, as well as various changes in liver cells [4, 5].

Flavin-containing monooxygenases (FMOs) are enzymes specializing in the oxidation of xeno-substrates. There is increasing evidence that a specific FMO gene is transcriptionally activated in numerous mouse longevity models, including mice treated with calorie restriction (CR), rapamycin, and growth hormone receptor knockout [6–8]. Moreover, it has been reported that activation of intestinal FMO2 induced by CR promoted longevity and health span [9]. The correlation between FMO overexpression and longevity suggests that FMOs could have a role in promoting health and longevity.

According to published data, FMO3 mRNA levels are markedly increased under 40% CR [10]. A microarray experiment has also detected highly expression level of FMO3 gene in CR mice and a positive correlation between FMO3 and lifespan has been remarked [11]. CR, defined as a nutritional regimen of reduced calorie intake without malnutrition, is considered to be one of the most robust interventions to delay the progression of aging and the development of age-associated alterations [12]. In addition, CR at 20% and 40% has been shown to significantly extend healthspan, particularly with respect to improvement of age-related alterations such as disordered hepatic fat metabolism [13–15]. These results suggest a close correlation of FMO3 with liver aging.

FMO3 is a protein of 532 amino acids, mainly expressed in the liver, where it contributes to drug biotransformation. Many oxidation reactions previously found to be catalyzed by cytochrome P450 enzymes were later determined to be catalyzed solely or predominately by FMO3, which may be responsible for about 6% of all phase I metabolic reactions [16]. However, there are limited data on the role of FMO3 in retarding hepatic aging. No published research, to date, has determined whether FMO3 overexpression alone exerts an anti-aging effect on the liver.

The induction of autophagy, a vital mechanism to promote cellular survival, is required for lifespan or healthspan extension in response to CR [17]. Nevertheless, the link between FMO3 and autophagy remains unknown. Further, several pathways shown to be involved in imparting the beneficial effects associated with CR have common signaling cascades and might coincide in their effects [13]. Thus, we chose to focus on some of the main mechanisms proposed for the anti-aging effects of CR, including increased autophagy. Moreover, as the mTOR signaling pathway is among the pathways by which CR is traditionally thought to induce autophagy, we aimed to elucidate the mechanism by which the upregulation of FMO3 retards liver aging by investigating the molecular interplay between FMO3 and mTOR-regulated autophagy.

In this study, we showed that FMO3 was upregulated by 40% CR, and the overexpression of FMO3 mimicked CR effects on alleviating many age-associated alterations, including amelioration of the serum and hepatic parameters related to inflammation, oxidative stress, lipid metabolism, liver function, and hepatocyte senescence. In addition, the inhibition of mTOR-regulated autophagy by Bafilomycin A1 suppressed the positive effects of FMO3 overexpression on liver aging. Overall, our results indicated that the upregulation of FMO3 reversed liver aging by inducing mTOR-regulated autophagy, which mimicked the effects of CR.

## RESULTS

### CR delays age-related alterations in aging liver

**Figure 1:**
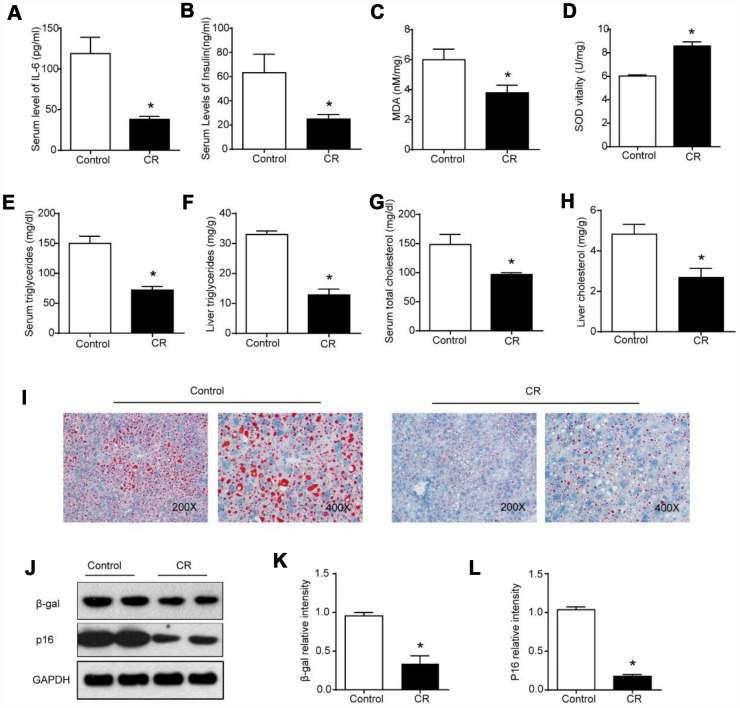
**40% CR for 6 months (14 to 20 months old) delays age-related alterations in aging liver.** Levels of (**A**) IL-6 and (**B**) insulin in serum were measured by ELISA. Levels of (**C**) MDA, (**D**) SOD, (**E**) serum TG, (**F**) liver TG, (**G**) serum TC, and (**H**) liver TC were measured using commercial kits. (**I**) Lipid droplet accumulation was assessed via Oil Red O staining. (**J**) Western blotting was used to determine levels of senescence markers (**K**) β-gal and (**L**) p16. Results are shown as the mean ± SD of eight animals per group. *p < 0.05 compared with the control group.

CR is considered to be the most successful anti-aging intervention [18]. We initially analyzed the effects of CR on whole-body and liver aging. According to previous studies, IL-6 levels and fasting insulin contents are elevated during aging [19, 20]. We observed lower serum levels of IL-6, suggesting moderate inflammation and immune response (Figure 1A), and lower fasting insulin levels in serum, indicating the amelioration of insulin resistance, in the CR group compared with the ad libitum-fed (AL) group (Figure 1B). As some studies have suggested that oxidative stress might play a part in the process of aging [21], markers reflecting hepatic oxidative damage or changes in the antioxidant system in the liver were measured. As shown in Figure 1C, 1D, the CR intervention reduced hepatic levels of malondialdehyde (MDA) and increased superoxidase dismutase (SOD) activity in the liver. It has been reported that changes in lipidomic and metabolomic signatures in the liver resulting from CR could contribute to the age-sparing effects of CR [22]. In our study, marked reductions in triglyceride (TG) and total cholesterol (TC) levels in serum and liver were observed after the CR intervention (Figure 1E–1H). We also performed Oil Red O staining to measure hepatic lipid contents. Histological analysis of liver specimens revealed that CR substantially decreased the accumulation of lipid droplets in the aging liver (Figure 1I). Previous studies of anti-aging interventions have indicated that β-gal (β-galactosidase) and p16 levels could serve as molecular markers of senescence [23]. As shown in Figure 1J–1L, the downregulation of β-gal and p16 confirmed the significant anti-aging effects of CR on the liver.

### CR upregulates levels of FMO3 in liver and enhances mTOR-regulated autophagy

**Figure 2:**
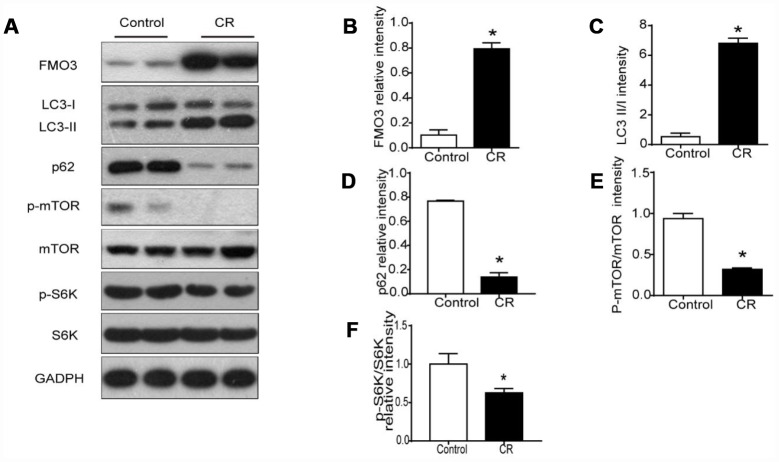
**40% CR for 6 months (14 to 20 months old) upregulates levels of FMO3 in liver and enhances mTOR-regulated autophagy.** (**A**) Representative western blots of liver lysates from each group of mice, probed with the indicated antibodies. GADPH was used as the internal housekeeping protein control. Image analysis of (**B**) FMO3, (**C**) LC3, (**D**) p62, (**E**) p-mTOR/mTOR, and (**F**) p-S6K/S6K was performed using densitometry. Results are shown as the mean ± SD of eight animals per group. *p < 0.05 compared with the control group.

Previous studies have shown markedly elevated levels of hepatic FMO3 mRNA in 40% CR mice [10]. Here, we investigated protein levels of FMO3 in liver after treatment with CR. As shown in Figure 2A, 2B, protein expression of FMO3 after CR was enhanced.

It has been well established that autophagy has a central role in the process of aging, as well as in all forms and at all stages of chronic liver disease [24]. Therefore, we analyzed LC3 and p62 to confirm whether the anti-aging effects of CR were modulated by autophagy. In CR mice, the LC3-II/I ratio increased significantly, while the level of p62 decreased, suggesting that CR promotes autophagy (Figure 2A, 2C, 2D). Given the critical role of mTOR signaling in autophagy, we examined whether CR influenced the activation of mTOR signaling. Our results showed that CR drastically diminished the phosphorylation of mTOR and the downstream S6K, indicating that mTOR signaling is involved in CR-induced autophagy (Figure 2A, 2E, 2F).

### Overexpression of FMO3 alleviates age-related alterations in aging liver

**Figure 3:**
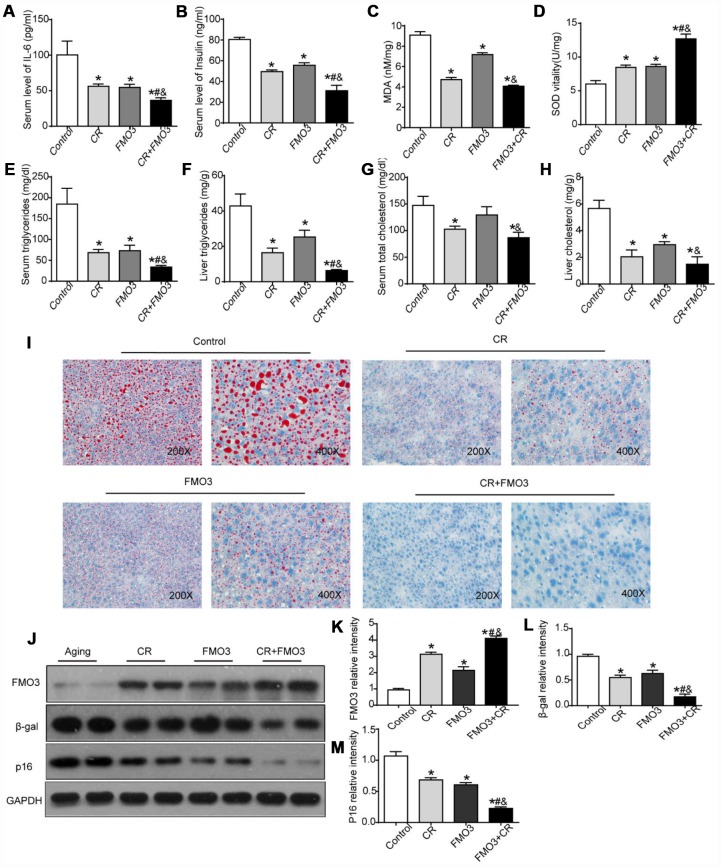
**Overexpression of FMO3 alleviates age-related alterations in aging liver.** Levels of (**A**) IL-6 and (**B**) insulin in serum were measured by ELISA. Levels of (**C**) SOD, (**D**) MDA, (**E**) serum TG, (**F**) liver TG, (**G**) serum TC, and (**H**) liver TC were measured using commercial kits. (**I**) Lipid droplet accumulation was assessed via Oil Red O staining. (**J**) Western blotting was used to determine levels of (**K**) FMO3, (**L**) β-gal, and (**M**) p16. Results are shown as the mean ± SD of eight animals per group. *p < 0.05 compared with the control group; ^#^p < 0.05 compared with the CR group; ^&^p < 0.05 compared with the FMO3 overexpression group.

To study the effects of FMO3 in aging mice, we used an adenovirus vector (Adv-FMO3) to activate FMO3 genes. The adenovirus vector was non-specific. The effects of CR, FMO3 overexpression, and their co-treatment were evaluated. Our studies showed that serum levels of IL-6 and insulin were significantly reduced in both the CR and FMO3 overexpression groups compared with the AL group. Combination treatment resulted in a marked decrease in serum IL-6 and insulin levels compared with the single treatments or control (Figure 3A, 3B). Next, we investigated the effects of CR, FMO3 overexpression, and their co-treatment on oxidative stress by measuring relative hepatic MDA and SOD levels. Hepatic MDA levels dramatically decreased and hepatic SOD levels drastically increased in the CR and FMO3 overexpression groups compared with the control group. The co-treatment groups showed less MDA activity and higher SOD levels compared with the single treatments or control (Figure 3C, 3D). Serum and liver lipid profiles showed that TG and TC levels in both serum and liver were markedly reduced after treatment with CR, FMO3 overexpression, and combination treatment. The co-treatment group also showed lower serum and hepatic TG and TC levels than the single treatment groups (Figure 3E–3H). Consistent with the TG and TC measurements, Oil Red O staining of the liver specimens showed a lower level of hepatic lipid accumulation in the co-treatment group compared with the other three groups, indicating a synergistic effect between FMO3 overexpression and CR. FMO3 and CR both substantially suppressed the accumulation of lipid droplets in aging liver (Figure 3I). Western blotting showed that the adenovirus expressing FMO3 (Ad-FMO3) caused robust overexpression of FMO3 in the liver (Figure 3J, 3K). Western blot analysis of the molecular markers of senescence revealed that FMO3 overexpression diminished levels of β-gal and p16, thus mimicking CR treatment. A synergistic effect was also observed in the co-treatment group (Figure 3J, 3L, 3M). Thus, FMO3 overexpression improved or reversed key serum and hepatic parameters related to inflammation, metabolism, oxidative stress, liver function, and hepatocyte senescence.

### FMO3 overexpression inhibits mTOR signaling and induces autophagy

**Figure 4:**
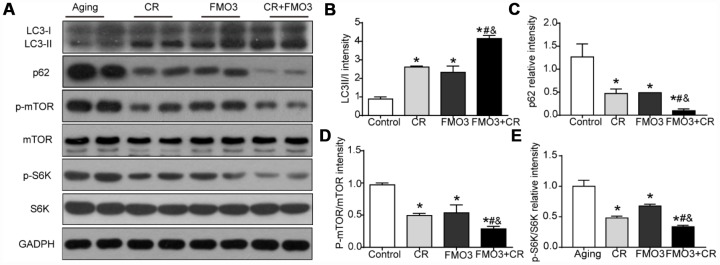
**FMO3 overexpression inhibits mTOR signaling and induces autophagy.** (**A**) Representative western blots of liver lysates from each group of mice, probed with the indicated antibodies. GADPH was used as the internal housekeeping protein control. Image analysis of (**B**) LC3, (**C**) p62, (**D**) p-mTOR/mTOR, and (**E**) p-S6K/S6K was performed using densitometry. Results are shown as the mean ± SD of eight animals per group. *p < 0.05 compared with the control group; ^#^p < 0.05 compared with the CR group; ^&^p < 0.05 compared with the FMO3 overexpression group.

To identify the mechanism of the protective effect of FMO3, we evaluated the level of autophagy and the activation of the upstream mTOR signaling that negatively regulated autophagy. As shown in Figure 4A–4C, the LC3-II/I ratio increased considerably and p62 content was greatly reduced in the CR, FMO3 overexpression, and co-treatment groups. The co-treatment group exhibited a higher LC3-II/I ratio and lower p62 level than the single treatment groups. These results indicate that FMO3 overexpression can augment autophagy and has a synergistic effect with CR. As shown in Figure 4A, 4D, 4E, FMO3 and CR both suppressed the phosphorylation of mTOR as well as the downstream S6K, and the combination treatment diminished the phosphorylation of mTOR and S6K substantially compared with single treatments or control. These results indicate that the upregulation of FMO3 acts synergistically with CR, suppressing the mTOR pathway and enhancing autophagy.

### Bafilomycin A1 treatment suppresses mTOR-regulated autophagy

**Figure 5:**
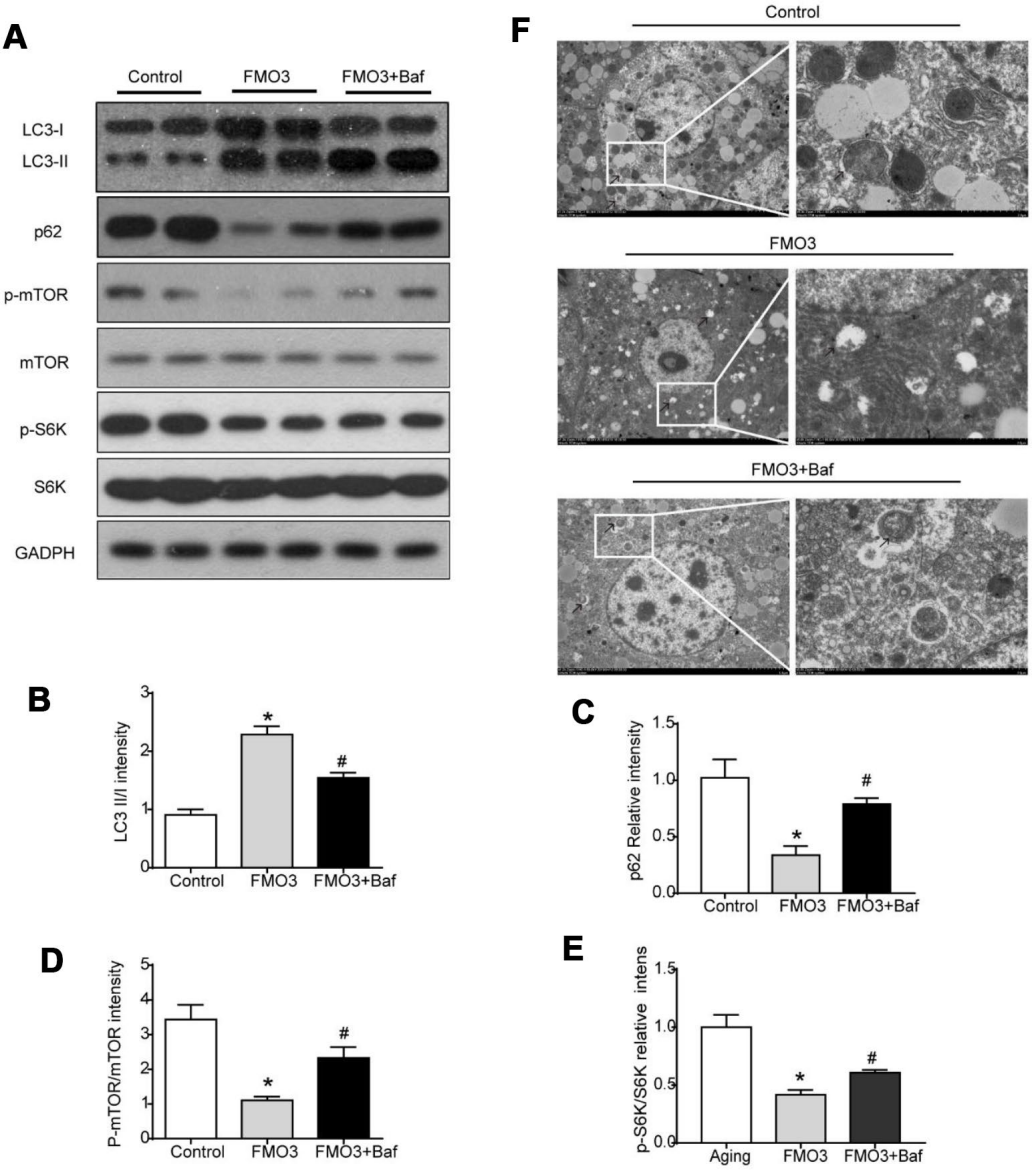
**Bafilomycin A1 treatment suppresses mTOR-regulated autophagy.** (**A**) Representative western blots of liver lysates from each group of mice, probed with the indicated antibodies. GADPH was used as the internal housekeeping protein control. Image analysis of (**B**) LC3, (**C**) p62, (**D**) p-mTOR/ mTOR, and (**E**) p-S6K/S6K was performed using densitometry. (**F**) Autophagosomes were observed through an electron microscope (black arrows). Results are shown as the mean ± SD of eight animals per group. *p < 0.05 compared with the control group; ^#^p < 0.05 compared with the FMO3 overexpression group.

To determine the role of autophagy specifically in the progress of FMO3-induced anti-aging effects on the liver, mice in the FMO3 overexpression group were intraperitoneally injected with a late-stage autophagy inhibitor, bafilomycin A1. The results were consistent with those obtained previously. As shown in Figure 5A–5C, compared with the control group, the FMO3 overexpression group exhibited an increased LC3II/LC3I ratio and decreased p62 content. Bafilomycin A1 is a late-stage autophagy inhibitor that prevents fusion between autophagosomes and lysosomes. The accumulation of LC3II and p62 indicated that autophagy degradation was inhibited successfully. Consistent with this, the FMO3 overexpression group also showed an immense increase in the number of autophagosomes, whereas the group receiving FMO3 overexpression combined with bafilomycin A1 treatment showed more accumulated autophagosomes than the single treatment group, resulting from the inhibition of autophagy in the late stage, as shown in the transmission electron microscopy images in Figure 5F. Notably, compared with the control group, reduced phosphorylation of mTOR and S6K was observed in the FMO3 overexpression group. Treatment with bafilomycin A1, however, abolished the FMO3-induced effects on mTOR/S6K signaling (Figure 5A, 5D, 5E).

### Autophagy inhibition blocks FMO3-induced anti-aging effects on liver

**Figure 6:**
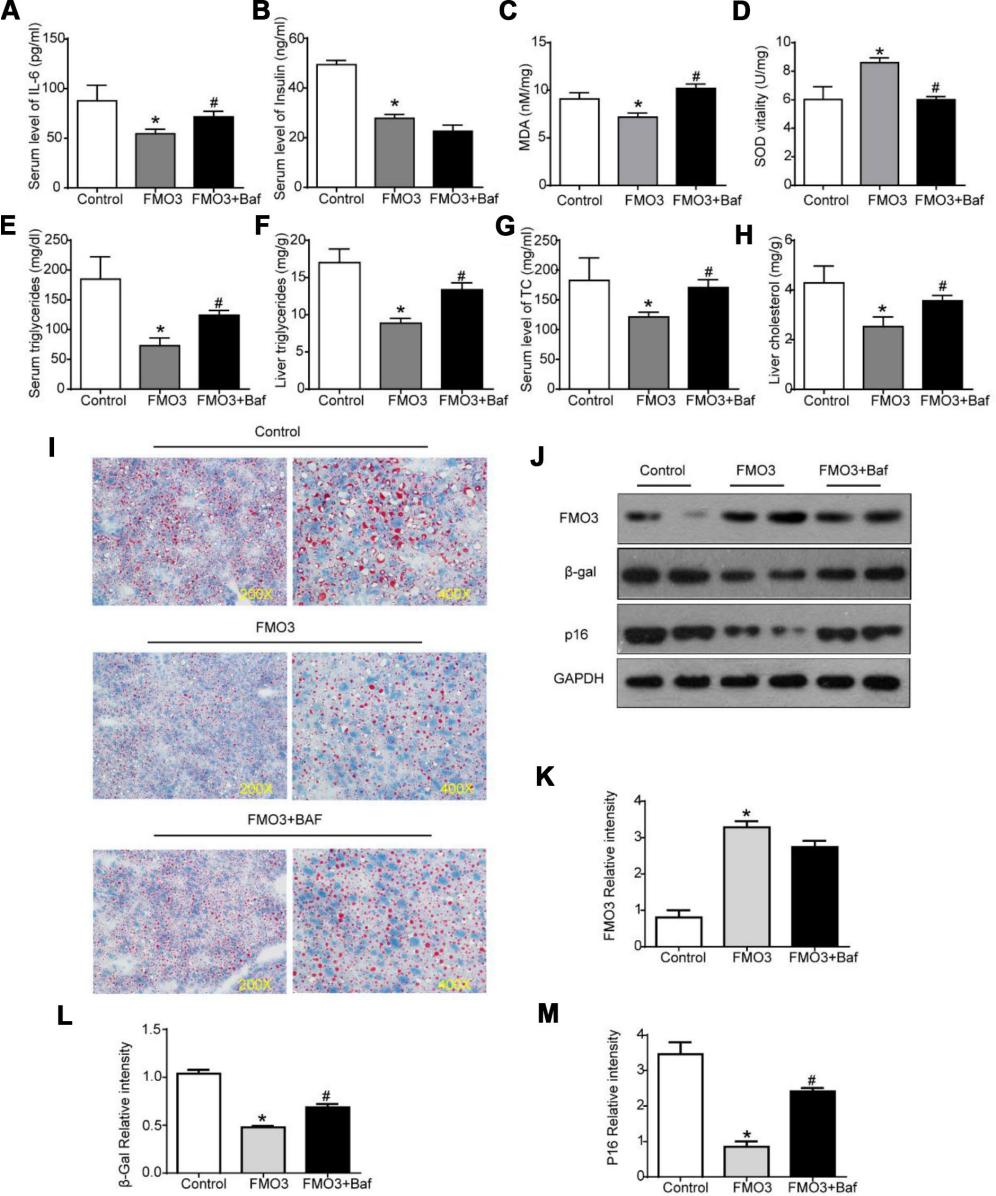
**Bafilomycin A1 treatment blocks FMO3-induced anti-aging effects on liver.** Levels of (**A**) IL-6 and (**B**) insulin in serum were measured by ELISA. Levels of (**C**) SOD, (**D**) MDA, (**E**) serum TG, (**F**) liver TG, (**G**) serum TC, and (**H**) liver TC were measured using commercial kits. (**I**) Lipid droplet accumulation was assessed via Oil Red O staining. (**J**) Western blotting was used to determine the levels of (**K**) FMO3, (**L**) β-gal, and (**M**) p16. Results are shown as the mean ± SD of eight animals per group. *p < 0.05 compared with the control group; ^#^p < 0.05 compared with the FMO3 overexpression group.

Next, we investigated whether autophagy inhibition by bafilomycin A1 treatment could reverse the FMO3-induced beneficial effects on the liver. As shown in Figure 6A, there was a conspicuous increase in serum levels of IL-6 in mice treated with both Ad-FMO3 and bafilomycin A1 compared with those treated with Ad-FMO3 alone. Serum levels of insulin, however, showed no significant changes after autophagy inhibition (Figure 6B). Mouse livers in the autophagy inhibition group exhibited markedly increased MDA and reduced SOD levels compared with the FMO3 overexpression group, indicating that bafilomycin A1 reversed the protective effect of FMO3 overexpression on oxidative stress in the liver (Figure 6C, 6D). As shown in Figure 6E–6H, bafilomycin A1 treatment greatly increased TG and TC levels in both serum and liver. Histologically, a notable reversion of the FMO3-induced reduction of lipid accumulation in the liver was also observed (Figure 6I) through Oil Red O staining of liver tissue. According to the western blot analysis, there were no significant differences in FMO3 protein expression levels between the groups with and without bafilomycin A1 treatment (Figure 6J, 6K). Levels of β-gal and p16 greatly increased following inhibition of autophagy by bafilomycin A1, compared with the FMO3 overexpression group without autophagy inhibition (Figure 6J, 6L–6N). Thus, the inhibition of autophagy abrogated the beneficial effects of the FMO3 overexpression intervention.

## DISCUSSION

Aging is usually accompanied by an increasing prevalence of age-related diseases. The implementation of anti-aging interventions is of great value for healthcare by virtue of its potential to prevent the vast majority of degenerative disorders before their age-related pathological manifestations occur [25]. CR is, to date, one of the most robust ways to retard the aging process of most organs, including the liver [12, 26]. However, in the long term, CR does not seem to be readily applicable to the majority of modern people. Hence, we aimed to investigate a novel therapeutic target for alternative treatment that mimics the beneficial effects of CR without the need for a long-term intervention involving food intake. Our results suggest, for the first time, the activation of FMO3 gene expression as a potential therapeutic approach mimicking the anti-aging effects of CR on the liver.

Previous work has shown that FMO3 mRNA in the liver was upregulated dramatically in 30–40% CR mouse models [10]. Our study further found that protein levels of FMO3 were elevated significantly. However, to the best of our knowledge, there have been no published studies exploring the effects of the FMO3 overexpression alone. Thus, we upregulated FMO3 in liver to investigate whether FMO3 overexpression alone was able to mimic the CR anti-aging effects.

The incidence rates of lipid and glucose metabolic disorders increase with age and have been noted as characteristics of hepatic senescence, which involves a decline in hepatic function [27]. Our results showed that the overexpression of FMO3 ameliorated lipid and glucose metabolic disorders in the aging model to the same extent as CR. The overexpression of FMO3 reduced TG and TC levels both in serum and liver, and diminished lipid droplet accumulation in the liver as a result of improved hepatic function. It may also help prevent insulin resistance, based on the reduction of fasting serum insulin levels in the FMO3 overexpression group. Also, co-treatment with CR and FMO3 overexpression exerted a synergistic effect in the amelioration of lipid and glucose metabolic disorders.

To better understand the anti-aging effects of FMO3 upregulation, the well-established cell senescence biomarkers β-gal and p16 were examined. Conspicuously reduced levels of β-gal and p16 were observed in the FMO3 overexpression group, providing further strong evidence that FMO3 overexpression alone exerts substantial anti-aging effects on the liver. A stronger effect was observed in the co-treatment group, which also exhibited the synergistic anti-aging effects of CR and FMO3 overexpression on the liver. As well as being a marker used to evaluate cellular senescence, p16 is a controllable factor regulating the aging process. It has been reported that the ablation of p16 in murine cells can extend lifespan and ameliorate age-associated dysfunction [23]. Therefore, the attenuation of p16 may be, to some extent, a plausible mechanism for the anti-aging effect of FMO3 overexpression. Further study is required to ascertain the interaction between FMO3 and these molecules.

Aging is a complex and combinatory process that involves many changes, including inflammation and oxidative damage [28]. Previous studies have reported an augmentation of pro-inflammatory cytokines such as IL-6, a reduction of SOD, and an elevated level of MDA in mouse models of aging [29, 30]. To further investigate the effects of FMO3 overexpression and how these effects are exerted, we analyzed the levels of pro-inflammatory cytokine IL-6 and oxidative stress indicators SOD and MDA. The reduction in IL-6 levels indicated that the overexpression of FMO3 attenuates inflammation. Nevertheless, more research is required to determine the exact role of inflammation in the anti-aging process induced by FMO3 overexpression. SOD functions as an antioxidant defense enzyme, while the end product of lipid peroxidation, MDA, is measured to reflect the level of hepatic oxidative damage. A conspicuous increase in SOD levels and markedly reduced MDA levels were observed in the CR group, FMO3 overexpression group, and co-treatment group, suggesting that FMO3 overexpression treatment has a synergistic effect with CR treatment in attenuating oxidative damage.

Recently, downregulation of autophagy has been reported to be present in numerous physical anomalies, including glucose and lipid metabolic disorders and hepatic accumulation of lipids [31, 32]. Autophagy is also deemed to be involved in alleviating inflammatory responses and oxidative stress [33, 34]. Increasing evidence suggests that autophagy has a critical role in hepatic aging and hepatic age-associated changes [35], and that the induction of autophagy is a key mechanism in the anti-aging process in response to CR [36, 37]. However, the correlation between FMO3 and autophagy remains unknown. Hence, we analyzed the LC3-II:LC3-I ratio and p62 level to determine whether FMO3 induced autophagy. Autophagosome formation depends on the transition of LC3 from LC3-II to LC3-I, and p62 is a protein degraded by autophagy. Hence, the LC3-II:LC3-I ratio represents the initiation of autophagy, while the p62 level reflects its activation. Our results showed that FMO3 overexpression treatment, as well as CR treatment, increased the LC3-II:LC3-I ratio and decreased p62 levels, indicating that the overexpression of FMO3 induces autophagy. In order to further investigate the involvement of autophagy in the anti-aging effect of FMO3, we inhibited autophagy using an inhibitor of late-stage autophagy, bafilomycin A1. Intriguingly, the beneficial effects of FMO3 overexpression, including amelioration of lipid and glucose metabolic disorders, diminished accumulation of lipid in liver, alleviation of inflammation and oxidative stress, and improved parameters of senescence markers, were all significantly diminished after inhibition of autophagy, indicating that FMO3 overexpression exerts its hepatic anti-aging effect by inducing autophagy. Given that autophagy can be induced via the inhibition of mTOR signaling in response to nutrient and energy status [38], we measured levels of p-mTOR and mTOR using western blotting. The p-mTOR:mTOR ratio was significantly decreased after FMO3 overexpression treatment, suggesting that FMO3 overexpression represses the mTOR signaling pathway.

Our results suggest a vital role for mTOR-regulated autophagy in the process by which FMO3 overexpression exerts its anti-aging effects on liver; however, some other mechanism besides inducing autophagy could also be involved. FMO3 is a hepatic enzyme specializing in the oxidation of xeno-substrates. Hence, there is a possibility that FMO3 substrates or products also have a role in aging. One of the well-known functions of FMO3 is catalyzing the oxidation of trimethylamine (TMA) to trimethylamine-N-oxide (TMAO). Some studies suggest that the FMO3/TMAO pathway is correlated with diabetes-associated cardiovascular disease [39], and others indicate that the effects of FMO3 on modulating glucose, lipid, and inflammation are independent of TMA/TMAO metabolism [40, 41]. Our study did not investigate whether the catalytic activities of FMO3 were involved in the process of liver aging. To better understand the mechanism by which FMO3 exerts its anti-aging effects on the liver, further research is required to determine the role of FMO3 substrates or products.

In conclusion, according to our results, FMO3 overexpression mimics the anti-aging effects of CR treatment and exerts a synergistic anti-aging effect with CR treatment in the aging liver by promoting autophagy.

## MATERIALS AND METHODS

### Animal models

Twelve-month-old male inbred C57BL/6 mice (weighing 30–40 g) were purchased from the Experimental Animal Center of the Chinese People’s Liberation Army no. 4 Military Medical University. Animals were maintained on a 12:12 light:dark cycle with lights on at 8:00 am and were fed a 20.5% protein rodent diet (Puluteng Ltd., Shanghai, China). Before the experiment, food consumption and body weights were monitored weekly for all mice for at least 2 weeks. Once food consumption had stabilized, food weights were calculated for the CR cohort based on the average daily food consumption of AL controls. The mice were 14 months old when we began conducting CR. During the following 6 months, all mice in the AL group had unrestricted access to food. All mice in the CR group received a 20% reduction of food supply for the first week compared with their AL intake, followed by a 30% reduction for the second week; the 40% reduction was started in the third week and continued until the end of the experiments. The CR animals were fed at the beginning of the light cycle. All groups had unrestricted access to water. Mice in the FMO+ groups were injected with Ad-FMO3 (1×109 particles/mouse), and the other groups with Ad-GFP, via the tail vein 2 weeks before sacrifice. Mice in the autophagy groups received an intraperitoneal injection of bafilomycin A1 (2 μM/kg) 24 hours after the tail intravenous injection, while the others received the same volume of 0.9% NaCl. The mice were 20 months old when they were sacrificed. The animal studies were approved by the Committee on the Ethics of Animal Experiments of Shanghai Jiao Tong University Affiliated Sixth People’s Hospital.

### Construction of adenovirus vectors

Ad-FMO3 was constructed by Yazai Biotech Ltd. (Shanghai, China). Reverse transcription polymerase chain reaction was used to amplify the cDNA fragment coding for the mouse FMO3 gene. The recombinant adenoviral vector expressing FMO3 was prepared in HepG2 cells. The titer of adenoviral vectors was determined using the gradient dilution method. The prepared adenovirus was stored at −80 °C for subsequent experiments. 1×109 particles/mouse of adenovirus [42] were delivered via the tail vein two weeks before sacrifice.

### Biochemical examinations and enzyme linked immunosorbent assay (ELISA)

Levels of TC and TG in serum and liver tissue were measured using commercially available kits provided by Nanjing Jiancheng Institute of Biotechnology (Nanjing, China) according to the manufacturer’s instructions. Serum insulin and IL-6 were examined respectively using mouse insulin ELISA kits from AIS (Hong Kong, China) and mouse IL-6 ELISA kits from Biovendor (Czech Republic) following the manufacturer’s protocols.

### Assay of antioxidant markers

Liver specimens were homogenized in phosphate-buffered saline to prepare 10% liver homogenate; this was then centrifuged at 4 °C and 3000 rpm for 10 min, and the supernatant was collected for the assays. Hepatic MDA activity and SOD contents were detected by a spectrophotometric method using commercially available kits provided by Nanjing Jiancheng Institute of Biotechnology (Nanjing, China). All assays were performed according to the manufacturer’s instructions.

### Western blot analysis

Western blotting was performed using a standard protocol. The liver sample was homogenized and lysed with a lysis buffer (RIPA with protease and phosphatase inhibitor). Total protein concentrations were determined using bicinchoninic acid protein assay kits (Beyotime Biotechnology, China). Proteins were separated with equal amounts in the same location from each specimen by sodium dodecyl sulfate polyacrylamide gel electrophoresis and transferred onto polyvinylidene difluoride membranes. Thereafter, the membranes were blocked with 5% skim milk for 1 hour at room temperature and incubated overnight with the appropriate antibodies at 4 °C, followed by incubation with horseradish peroxidase-labeled secondary antibody (1:5000, Bio-Rad, USA) for 1 hour at room temperature. The primary antibodies included anti-FMO3 rabbit mAb (1:1000, Abcam, USA), mTOR rabbit mAb (1:1000, Cell Signaling Technology, USA), phospho-mTOR rabbit mAb (1:1000, Cell Signaling Technology), S6K rabbit mAb (1:1000, Cell Signaling Technology, USA), phospho-S6K rabbit mAb (1:1000, Cell Signaling Technology), p16 rabbit mAb (1:1000, Cell Signaling Technology), β-gal rabbit mAb (1:1000, Cell Signaling Technology), LC3II/I rabbit mAb (1:1000, Cell Signaling Technology), and P62 rabbit mAb (1:1000, Cell Signaling Technology). Finally, the immunoreacting bands were visualized by an enhanced chemiluminescence method using an electrochemiluminescent solution (Millipore, USA) and the ImageJ (NIH, USA) analysis system.

### Oil Red O assay

Frozen liver tissue was cut into 3-μm-thick sections, then stained with filtered Oil Red O (EMD Millipore) dissolved in 60% isopropanol for 15 min at room temperature. Afterwards, the slides were incubated in hematoxylin to counterstain the nuclei and transferred to an aqueous mounting medium (Thermo Fisher Scientific). Images were captured under a light microscope (magnification x200 and x400).

### Transmission electron microscopy

Liver samples were fixed in 3% glutaraldehyde at 4 °C for 24 hours, post-fixed in 1% osmium tetroxide in sodium phosphate buffer at room temperature, and cut into ultrathin sections (50–70 nm thick) with an ultramicrotome. Sections were stained with 2% uranyl acetate and lead citrate, then viewed and photographed using a Hitachi H-7650 transmission electron microscope at 120 kV. Ultrastructural analysis was performed using ImageJ (NIH).

### Statistical analysis

Sample sizes, as described in the figure legends, were selected based on effect size and availability, according to the usual standard using the “resource equation” method [43]. Statistical analysis was performed using the GraphPad Prism 7.0 software. Data are presented as mean ± standard deviation (SD). One-way analysis of variance was used to assess statistical significance using Tukey post hoc analysis; p-values less than 0.05 were considered to be statistically significant.

## References

[1] Pazoki-Toroudi H, Amani H, Ajami M, Nabavi SF, Braidy N, Kasi PD, Nabavi SM. Targeting mTOR signaling by polyphenols: A new therapeutic target for ageing. Ageing Res Rev. 2016; 31:55–66. 10.1016/j.arr.2016.07.004 27453478

[2] Kume S, Uzu T, Horiike K, Chin-Kanasaki M, Isshiki K, Araki S, Sugimoto T, Haneda M, Kashiwagi A, Koya D. Calorie restriction enhances cell adaptation to hypoxia through Sirt1-dependent mitochondrial autophagy in mouse aged kidney. J Clin Invest. 2010; 120:1043–55. 10.1172/JCI41376 20335657 PMC2846062

[3] Mao SA, Glorioso JM, Nyberg SL. Liver regeneration. Transl Res. 2014; 163:352–62. 10.1016/j.trsl.2014.01.005 24495569 PMC3976740

[4] Kim IH, Kisseleva T, Brenner DA. Aging and liver disease. Curr Opin Gastroenterol. 2015; 31:184–91. 10.1097/MOG.0000000000000176 25850346 PMC4736713

[5] Gokarn R, Solon-Biet S, Youngson NA, Wahl D, Cogger VC, McMahon AC, Cooney GJ, Ballard JW, Raubenheimer D, Morris MJ, Simpson SJ, Le Couteur DG. The Relationship Between Dietary Macronutrients and Hepatic Telomere Length in Aging Mice. J Gerontol A Biol Sci Med Sci. 2018; 73:446–49. 10.1093/gerona/glx186 30052781

[6] Swindell WR. Genes and gene expression modules associated with caloric restriction and aging in the laboratory mouse. BMC Genomics. 2009; 10:585. 10.1186/1471-2164-10-585 19968875 PMC2795771

[7] Steinbaugh MJ, Sun LY, Bartke A, Miller RA. Activation of genes involved in xenobiotic metabolism is a shared signature of mouse models with extended lifespan. Am J Physiol Endocrinol Metab. 2012; 303:E488–95. 10.1152/ajpendo.00110.2012 22693205 PMC3423099

[8] Miller RA, Harrison DE, Astle CM, Fernandez E, Flurkey K, Han M, Javors MA, Li X, Nadon NL, Nelson JF, Pletcher S, Salmon AB, Sharp ZD, et al. Rapamycin-mediated lifespan increase in mice is dose and sex dependent and metabolically distinct from dietary restriction. Aging Cell. 2014; 13:468–77. 10.1111/acel.12194 24341993 PMC4032600

[9] Leiser SF, Miller H, Rossner R, Fletcher M, Leonard A, Primitivo M, Rintala N, Ramos FJ, Miller DL, Kaeberlein M. Cell nonautonomous activation of flavin-containing monooxygenase promotes longevity and health span. Science. 2015; 350:1375–78. 10.1126/science.aac9257 26586189 PMC4801033

[10] Fu ZD, Klaassen CD. Short-term calorie restriction feminizes the mRNA profiles of drug metabolizing enzymes and transporters in livers of mice. Toxicol Appl Pharmacol. 2014; 274:137–46. 10.1016/j.taap.2013.11.003 24240088 PMC4659498

[11] Swindell WR. Gene expression profiling of long-lived dwarf mice: longevity-associated genes and relationships with diet, gender and aging. BMC Genomics. 2007; 8:353. 10.1186/1471-2164-8-353 17915019 PMC2094713

[12] López-Lluch G, Navas P. Calorie restriction as an intervention in ageing. J Physiol. 2016; 594:2043–60. 10.1113/JP270543 26607973 PMC4834802

[13] Derous D, Mitchell SE, Wang L, Green CL, Wang Y, Chen L, Han JJ, Promislow DE, Lusseau D, Douglas A, Speakman JR. The effects of graded levels of calorie restriction: XI. Evaluation of the main hypotheses underpinning the life extension effects of CR using the hepatic transcriptome. Aging (Albany NY). 2017; 9:1770–824. 10.18632/aging.101269 28768896 PMC5559174

[14] Mitchell SJ, Madrigal-Matute J, Scheibye-Knudsen M, Fang E, Aon M, González-Reyes JA, Cortassa S, Kaushik S, Gonzalez-Freire M, Patel B, Wahl D, Ali A, Calvo-Rubio M, et al. Effects of Sex, Strain, and Energy Intake on Hallmarks of Aging in Mice. Cell Metab. 2016; 23:1093–112. 10.1016/j.cmet.2016.05.027 27304509 PMC4911707

[15] Wahl D, Solon-Biet SM, Wang QP, Wali JA, Pulpitel T, Clark X, Raubenheimer D, Senior AM, Sinclair DA, Cooney GJ, de Cabo R, Cogger VC, Simpson SJ, Le Couteur DG. Comparing the Effects of Low-Protein and High-Carbohydrate Diets and Caloric Restriction on Brain Aging in Mice. Cell Rep. 2018; 25:2234–2243.e6. 10.1016/j.celrep.2018.10.070 30463018 PMC6296764

[16] Xu M, Bhatt DK, Yeung CK, Claw KG, Chaudhry AS, Gaedigk A, Pearce RE, Broeckel U, Gaedigk R, Nickerson DA, Schuetz E, Rettie AE, Leeder JS, et al. Genetic and Nongenetic Factors Associated with Protein Abundance of Flavin-Containing Monooxygenase 3 in Human Liver. J Pharmacol Exp Ther. 2017; 363:265–74. 10.1124/jpet.117.243113 28819071 PMC5697103

[17] Nacarelli T, Azar A, Sell C. Aberrant mTOR activation in senescence and aging: A mitochondrial stress response? Exp Gerontol. 2015; 68:66–70. 10.1016/j.exger.2014.11.004 25449851 PMC4589173

[18] Escobar KA, Cole NH, Mermier CM, VanDusseldorp TA. Autophagy and aging: maintaining the proteome through exercise and caloric restriction. Aging Cell. 2019; 18:e12876. 10.1111/acel.12876 30430746 PMC6351830

[19] Daynes RA, Araneo BA, Ershler WB, Maloney C, Li GZ, Ryu SY. Altered regulation of IL-6 production with normal aging. Possible linkage to the age-associated decline in dehydroepiandrosterone and its sulfated derivative. J Immunol. 1993; 150:5219–30. 8515056

[20] Du W, Wong C, Song Y, Shen H, Mori D, Rotllan N, Price N, Dobrian AD, Meng H, Kleinstein SH, Fernandez-Hernando C, Goldstein DR. Age-associated vascular inflammation promotes monocytosis during atherogenesis. Aging Cell. 2016; 15:766–77. 10.1111/acel.12488 27135421 PMC4933655

[21] Yew WW, Chang KC, Chan DP. Oxidative Stress and First-Line Antituberculosis Drug-Induced Hepatotoxicity. Antimicrob Agents Chemother. 2018; 62. 10.1128/AAC.02637-17 29784840 PMC6105810

[22] Jové M, Naudí A, Ramírez-Núñez O, Portero-Otín M, Selman C, Withers DJ, Pamplona R. Caloric restriction reveals a metabolomic and lipidomic signature in liver of male mice. Aging Cell. 2014; 13:828–37. 10.1111/acel.12241 25052291 PMC4331741

[23] Niedernhofer LJ, Kirkland JL, Ladiges W. Molecular pathology endpoints useful for aging studies. Ageing Res Rev. 2017; 35:241–49. 10.1016/j.arr.2016.09.012 27721062 PMC5357461

[24] Flores-Toro JA, Go KL, Leeuwenburgh C, Kim JS. Autophagy in the liver: cell’s cannibalism and beyond. Arch Pharm Res. 2016; 39:1050–61. 10.1007/s12272-016-0807-8 27515049 PMC5007189

[25] Kennedy BK, Pennypacker JK. Drugs that modulate aging: the promising yet difficult path ahead. Transl Res. 2014; 163:456–65. 10.1016/j.trsl.2013.11.007 24316383 PMC4004650

[26] Sato S, Solanas G, Peixoto FO, Bee L, Symeonidi A, Schmidt MS, Brenner C, Masri S, Benitah SA, Sassone-Corsi P. Circadian Reprogramming in the Liver Identifies Metabolic Pathways of Aging. Cell. 2017; 170:664–677.e11. 10.1016/j.cell.2017.07.042 28802039 PMC7792549

[27] He J, Zheng H, Pan D, Liu T, Sun Y, Cao J, Wu Z, Zeng X. Effects of aging on fat deposition and meat quality in Sheldrake duck. Poult Sci. 2018; 97:2005–10. 10.3382/ps/pey077 29554357

[28] Bullone M, Lavoie JP. The Contribution of Oxidative Stress and Inflamm-Aging in Human and Equine Asthma. Int J Mol Sci. 2017; 18:2612. 10.3390/ijms18122612 29206130 PMC5751215

[29] Kim IH, Xu J, Liu X, Koyama Y, Ma HY, Diggle K, You YH, Schilling JM, Jeste D, Sharma K, Brenner DA, Kisseleva T. Aging increases the susceptibility of hepatic inflammation, liver fibrosis and aging in response to high-fat diet in mice. Age (Dordr). 2016; 38:291–302. 10.1007/s11357-016-9938-6 27578257 PMC5061686

[30] Jimenez AG, Winward J, Beattie U, Cipolli W. Cellular metabolism and oxidative stress as a possible determinant for longevity in small breed and large breed dogs. PLoS One. 2018; 13:e0195832. 10.1371/journal.pone.0195832 29694441 PMC5918822

[31] Marasco MR, Linnemann AK. β-Cell Autophagy in Diabetes Pathogenesis. Endocrinology. 2018; 159:2127–41. 10.1210/en.2017-03273 29617763 PMC5913620

[32] Zhang Y, Sowers JR, Ren J. Targeting autophagy in obesity: from pathophysiology to management. Nat Rev Endocrinol. 2018; 14:356–76. 10.1038/s41574-018-0009-1 29686432

[33] Nurmi K, Kareinen I, Virkanen J, Rajamäki K, Kouri VP, Vaali K, Levonen AL, Fyhrquist N, Matikainen S, Kovanen PT, Eklund KK. Hemin and Cobalt Protoporphyrin Inhibit NLRP3 Inflammasome Activation by Enhancing Autophagy: A Novel Mechanism of Inflammasome Regulation. J Innate Immun. 2017; 9:65–82. 10.1159/000448894 27655219 PMC6738905

[34] Medvedev R, Hildt E, Ploen D. Look who’s talking-the crosstalk between oxidative stress and autophagy supports exosomal-dependent release of HCV particles. Cell Biol Toxicol. 2017; 33:211–31. 10.1007/s10565-016-9376-3 27987184

[35] Czaja MJ. Autophagy in health and disease. 2. Regulation of lipid metabolism and storage by autophagy: pathophysiological implications. Am J Physiol Cell Physiol. 2010; 298:C973–78. 10.1152/ajpcell.00527.2009 20089934 PMC2867392

[36] He LQ, Lu JH, Yue ZY. Autophagy in ageing and ageing-associated diseases. Acta Pharmacol Sin. 2013; 34:605–11. 10.1038/aps.2012.188 23416930 PMC3647216

[37] Donati A, Recchia G, Cavallini G, Bergamini E. Effect of aging and anti-aging caloric restriction on the endocrine regulation of rat liver autophagy. J Gerontol A Biol Sci Med Sci. 2008; 63:550–55. 10.1093/gerona/63.6.550 18559627

[38] Xu L, Brink M. mTOR, cardiomyocytes and inflammation in cardiac hypertrophy. Biochim Biophys Acta. 2016; 1863:1894–903. 10.1016/j.bbamcr.2016.01.003 26775585

[39] Miao J, Ling AV, Manthena PV, Gearing ME, Graham MJ, Crooke RM, Croce KJ, Esquejo RM, Clish CB, Vicent D, Biddinger SB, and Morbid Obesity Study Group. Flavin-containing monooxygenase 3 as a potential player in diabetes-associated atherosclerosis. Nat Commun. 2015; 6:6498. 10.1038/ncomms7498 25849138 PMC4391288

[40] Shih DM, Wang Z, Lee R, Meng Y, Che N, Charugundla S, Qi H, Wu J, Pan C, Brown JM, Vallim T, Bennett BJ, Graham M, et al. Flavin containing monooxygenase 3 exerts broad effects on glucose and lipid metabolism and atherosclerosis. J Lipid Res. 2015; 56:22–37. 10.1194/jlr.M051680 25378658 PMC4274068

[41] Warrier M, Shih DM, Burrows AC, Ferguson D, Gromovsky AD, Brown AL, Marshall S, McDaniel A, Schugar RC, Wang Z, Sacks J, Rong X, Vallim TA, et al. The TMAO-Generating Enzyme Flavin Monooxygenase 3 Is a Central Regulator of Cholesterol Balance. Cell Rep. 2015; 10:326–38. 10.1016/j.celrep.2014.12.036 25600868 PMC4501903

[42] Gong Q, Hu Z, Zhang F, Cui A, Chen X, Jiang H, Gao J, Chen X, Han Y, Liang Q, Ye D, Shi L, Chin YE, et al. Fibroblast growth factor 21 improves hepatic insulin sensitivity by inhibiting mammalian target of rapamycin complex 1 in mice. Hepatology. 2016; 64:425–38. 10.1002/hep.28523 26926384 PMC5726522

[43] Charan J, Kantharia ND. How to calculate sample size in animal studies? J Pharmacol Pharmacother. 2013; 4:303–06. 10.4103/0976-500X.119726 24250214 PMC3826013

